# Comparison Between Alpelisib Plus Endocrine Therapy and Everolimus Plus Endocrine Therapy After CDK4/6 Inhibitors Progression in Patients with *PIK3CA*-Mutant Metastatic Breast Cancer: A Single-Center Retrospective Study

**DOI:** 10.3390/cancers18030468

**Published:** 2026-01-30

**Authors:** Sotiris Loizidis, Damianos Michaelides, Yiola Marcou, Eleni Kakouri, Ifigenia Konstantinou, Anastasia Constantinidou, Stavroula Theophanous-Kitiri, Elisavet Papageorgiou, Kyriaki Michailidou, Myria Galazi

**Affiliations:** 1 Department of Medical Oncology, Bank of Cyprus Oncology Center, 32 Acropoleos Avenue, Nicosia 2006, Cyprus; yiola.marcou@bococ.org.cy (Y.M.); eleni.kakouri@bococ.org.cy (E.K.); ifigenia.konstantinou@bococ.org.cy (I.K.); anastasia.constantinidou@bococ.org.cy (A.C.); myria.galazi@bococ.org.cy (M.G.); 2 Department of Medical Oncology, German Medical Institute, 1 Nikis Avenue, Limassol 4108, Cyprus; 3 Biostatistics Unit, The Cyprus Institute of Neurology and Genetics, 6 Iroon Avenue, Nicosia 2371, Cyprus; damianosm@cing.ac.cy (D.M.); kyriakimi@cing.ac.cy (K.M.); 4 School of Medicine, University of Cyprus, Panepistimiou 1, Nicosia 2408, Cyprus; 5 Pharmacy Department, Bank of Cyprus Oncology Center, 32 Acropoleos Avenue, Nicosia 2006, Cyprus; 6 A.G. Leventis Clinical Trials Unit, Bank of Cyprus Oncology Center, 32 Acropoleos Avenue, Nicosia 2006, Cyprus; elisavet.papageorgiou@bococ.org.cy

**Keywords:** breast cancer, targeted therapies, alpelisib, everolimus, endocrine therapy, precision medicine

## Abstract

**Background**: Evidence on the efficacy of alpelisib in combination with fulvestrant after progression on CKD4/6 inhibitors (CDK4/6i) is derived from a single non-comparative prospective study. Conversely, the effectiveness of everolimus plus exemestane on *PIK3CA*-mutant metastatic breast cancer (BC) after CDK4/6i failure has never been investigated in a prospective study. In this retrospective study, we compared alpelisib plus endocrine therapy (ET) with everolimus plus exemestane in patients with *PIK3CA*-mutant metastatic BC post-CDK4/6i progression. **Methods**: We tracked 40 patients treated with alpelisib plus ET and 22 treated with everolimus plus exemestane. We further identified 42 patients who did not harbor *PIK3CA* mutations (*PIK3CA*-wild-type group) and received everolimus as a subsequent treatment after progression on CDK4/6i. The timeframe spanned from 1st March 2020 to 30th November 2024. **Results**: The median progression-free survival (PFS) for the alpelisib group was 4.9 months compared to 4.5 months for the everolimus group [Hazard ratio (HR), 1.22; 95% CI, 0.65–2.28; *p*-value = 0.53]. The median overall survival (OS) was 9.6 months and 18.3 months for alpelisib and everolimus, respectively (HR, 0.67; 95% CI, 0.25–1.76; *p*-value = 0.47). Median PFS in the *PIK3CA*-mutant everolimus plus ET group was 4.5 months (95% CI, 2.8–6.7) compared to 5 months (95% CI, 3.5–6.9) for the *PIK3CA*-wild-type everolimus plus ET group (HR, 0.77; 95% CI, 0.46–1.29; *p*-value = 0.32). The most common side effects in the alpelisib group were hyperglycemia (57.5%), rash (27.5%), and anorexia (22.5%), while the most common side effects in the everolimus group were fatigue (40.9%) and stomatitis (27.3%). **Conclusions**: Our results regarding the efficacy of alpelisib plus ET were inferior to those reported in the current literature. Conversely, outcomes of everolimus plus exemestane were consistent with the current literature, denoting that the combination is an acceptable treatment option for patients with *PIK3CA*-mutant metastatic BC.

## Simple Summary

Alpelisib, an α-specific PI3K inhibitor, was approved as the first targeted therapy against *PIK3CA*-mutant metastatic breast cancer. Nevertheless, its efficacy post CDK4/6 inhibitors failure has not yet been tested in a randomized trial. Everolimus, an *mTOR* inhibitor, is still in use in many countries, but, like alpelisib, it has never been evaluated at a randomized trial level after CDK4/6 inhibitor failure in *PIK3CA*-mutant metastatic breast cancer. In this single-center retrospective study, we compare alpelisib plus endocrine therapy with everolimus plus endocrine therapy in patients with *PIK3CA*-mutant metastatic breast cancer who progressed on CDK4/6 inhibitors.

## 1. Introduction

Phosphoinositide 3-kinases (PI3Ks) are a group of lipid kinases that are part of the *PI3K*/*AKT*/*mTOR* signaling pathway. They play a role in mediating signals related to cell survival, differentiation, proliferation and metabolism under normal physiological conditions [1]. PI3Ks are classified into three main types (I–III), with class I further subdivided into class IA and IB based on their activators. Class IA PI3Ks are heterodimers that consist of a catalytic (p110) and a regulatory (p85) subunit [1,2]. The p110 catalytic subunit is encoded by the *PIK3CA* gene, which is among the most frequently mutated genes in breast cancer (BC) [1,3]. The incidence of *PIK3CA* mutations is higher in the luminal BC subtype, reaching up to 40%, while mutations in the basal and HER2-enriched subtypes are less common [3,4]. Of note, approximately 12% of *PIK3CA*-mutant BC harbor double *PIK3CA* mutations [5].

The unprecedented progress made through targeted therapies in metastatic HR-positive/HER2-negative (HR+/HER2−) BC has led to a significant improvement in survival and quality of life. Tumors harboring *PIK3CA* mutations exhibit a poor response to chemotherapy; nevertheless, targeted therapies, which inhibit the hyperactivated *PI3K*/*AKT*/*mTOR* pathway, have demonstrated remarkable benefit [6,7]. One such drug is the small molecule alpelisib, a potent α-specific PI3K inhibitor that selectively inhibits the p110α subunit. It was approved by the Food and Drug Administration (FDA) and the European Medicines Agency (EMA) in 2019 and 2020, respectively, based on the results of the randomized, phase III SOLAR-1 trial [8,9,10]. Three hundred and forty-one (341) patients diagnosed with metastatic *PIK3CA*-mutant HR+/HER2− BC (*PIK3CA*-mutant cohort) were randomly assigned to either the alpelisib plus fulvestrant arm or the placebo plus fulvestrant arm. The study demonstrated a statistically significant difference in terms of median PFS, favoring alpelisib by 11 months compared to 5.7 months for the placebo arm [Hazard ratio (HR), 0.65; 95% CI, 0.50–0.85; *p* < 0.001]. The most commonly reported adverse events of any grade were hyperglycemia (63.7%), diarrhea (57.7%) and nausea (44.7%). The final analysis of the SOLAR-1 study showed a median OS in the alpelisib group of 39.3 months compared to 31.4 months for the placebo arm. Despite the 7.9-month absolute difference, the endpoint did not cross the prespecified boundary for statistical significance (HR: 0.86, 95% CI, 0.64–1.15; *p* = 0.15) [10,11].

The positive results of the pivotal SOLAR-1 trial and the subsequent introduction of alpelisib have nonetheless raised new clinical questions. The patients in SOLAR-1 should have had prior exposure to aromatase inhibitors (AI), either in the (neo-)adjuvant or metastatic setting. A total of 52.1% of patients received alpelisib as first-line therapy, while the rest received it as second-line therapy. The previous use of cyclin-dependent kinase 4 and 6 inhibitors (CDK4/6i) was not a prerequisite, and only 5.3% of patients in the alpelisib arm received them. The subgroup analysis of the nine patients who had been exposed to CDK4/6i before alpelisib revealed median PFS and OS of 5.5 months and 29.8 months, respectively, compared with 1.8 months and 12.9 months for the placebo group [11].

Given that CDK4/6i in combination with endocrine therapy represent the undisputed first-line treatment option, the efficacy of alpelisib post-CDK4/6i failure remains to be determined by a randomized trial. Nevertheless, a glance at the effectiveness of alpelisib plus fulvestrant after disease progression on CDK4/6i was provided by the phase II, multicohort, non-comparative BYLieve study. Cohort A of the study enrolled 127 patients who experienced progression on CDK4/6i and subsequently received the combination. After a median follow-up of 21.8 months, the reported median PFS was 8 months, whilst the median OS was 27.3 months [12].

Everolimus is an oral *mTOR* inhibitor approved since 2012, long before the CDK4/6i era, for the treatment of metastatic BC as a subsequent therapy after progression on AIs, based on the results of the BOLERO-2 trial [13]. *mTOR* is a serine–threonine kinase and is part of the downstream *PI3K*/*AKT*/*mTOR* pathway. Therefore, it is plausible to hypothesize that inhibiting *mTOR* in a constitutively active *PI3K*/*AKT*/*mTOR* pathway could potentially halt the tumorigenic signaling. However, evidence regarding the impact of everolimus on *PIK3CA*-mutant BC is limited. An exploratory analysis of the BOLERO-2 trial showed that patients with *PIK3CA* mutations who received everolimus had a median PFS of 6.7 months [14]. Notably, this analysis was conducted before the era of CDK4/6i, while the efficacy of everolimus plus exemestane after failure of CDK4/6i remains largely unknown to this day. Vasseur et al. recently published the results of a prospective study in which patients were treated with everolimus plus fulvestrant after progression on CDK4/6i. A total of 25 out of 47 patients harbored *PIK3CA* mutations, and this subgroup exhibited median PFS and OS of 3.4 months and 22.9 months, respectively [15].

In this single-center retrospective study, we compared alpelisib plus ET versus everolimus plus ET in patients with *PIK3CA*-mutant metastatic HR+/HER2− BC whose disease progressed on CDK4/6i. Moreover, we provided insights about the real-world toxicity profile of alpelisib.

## 2. Materials and Methods

Based on our center’s electronic database, we retrospectively reviewed and identified patients diagnosed with metastatic HR+/HER2− BC harboring *PIK3CA* mutations who received CDK4/6i plus ET as first-line treatment and, after progression, were placed on alpelisib or everolimus (*PIK3CA*-mutant group) in combination with ET. Patients who started alpelisib and subsequently received everolimus were excluded from the survey. We further constituted another group of 42 patients who did not harbor *PIK3CA* mutations (*PIK3CA*-wild-type group) and received everolimus as a subsequent treatment after progression on CDK4/6i. The demographic data are listed in Table 1. The study timeframe was defined as 1 May 2020 to 30 November 2024, with the cut-off follow-up date set to 31 March 2025. The latter date was selected to enable at least four months of follow-up data. Forty (40) patients were identified and embedded in the alpelisib cohort, while 22 patients were included in the everolimus cohort (*PIK3CA*-mutant). Forty-two (42) patients who received everolimus were included in the *PIK3CA*-wild-type cohort. *PIK3CA* mutations were identified in tissue samples using the next-generation sequencing (NGS) Oncomine^TM^ Focus assay (Thermo Fisher Scientific, Waltham, MA, USA), and molecular analysis was performed at a central laboratory. Licensed circulating-tumor DNA (ctDNA) assays were not used. Most commonly detected mutations in the alpelisib arm were H1047R (40%), E545K (20%), and E542K (15%). All patients were followed and monitored at specific timepoints during the course of treatment and underwent imaging every three months. Radiological assessment was available for 29 patients in the alpelisib group and for 19 patients in the everolimus group (*PIK3CA*-mutant), respectively. Radiological assessment was executed according to Response Evaluation Criteria in Solid Tumors (RECIST) version 1.1. The study obtained local approval from the Bank of Cyprus research committee, which reviewed and approved the study protocol. The Cyprus National Bioethics Committee provided national approval.

### Statistical Analysis

Median survival times with corresponding 95% confidence intervals (CIs) for PFS and OS were assessed using the Kaplan–Meier method. PFS was defined as the time from initiation of treatment to disease progression or death from any cause. OS was defined as the time from initiation of therapy to the date of death from any cause. Patients who were alive as of the cutoff date (31st of March 2025) or were lost in follow-up were censored. Differences between cohorts were evaluated with the log-rank test. HRs and 95% CIs were estimated using Cox proportional hazards regression models. Objective response rates (ORRs) were estimated as the proportion of patients who achieved a partial response. Given the binary nature of the outcome, i.e., response or no response, responders were assumed to follow a binomial distribution. Additionally, 95% CIs for the ORRs were calculated using binomial proportion following the Clopper–Pearson method. Statistical analyses were performed using the software R (version 4.5.1). *p*-values ≤ 0.05 were considered statistically significant.

## 3. Results

### 3.1. Survival Analysis

**Figure 1:**
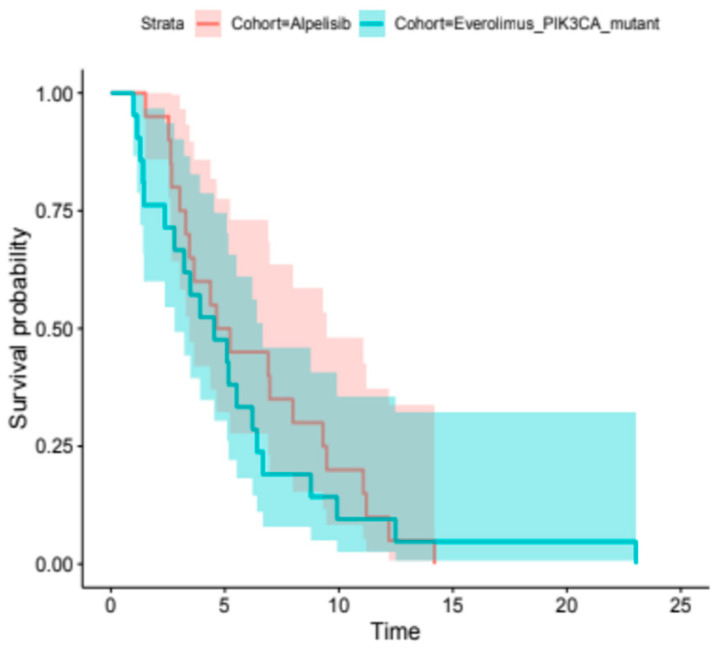
Kaplan–Meier analysis of PFS. The median PFS for the alpelisib + ET group was 4.9 months (95% CI, 3.4–9.5), whereas the median PFS was 4.5 months (95% CI, 2.8–6.7) for the everolimus plus ET group (log-rank, *p*-value = 0.53).

**Figure 2:**
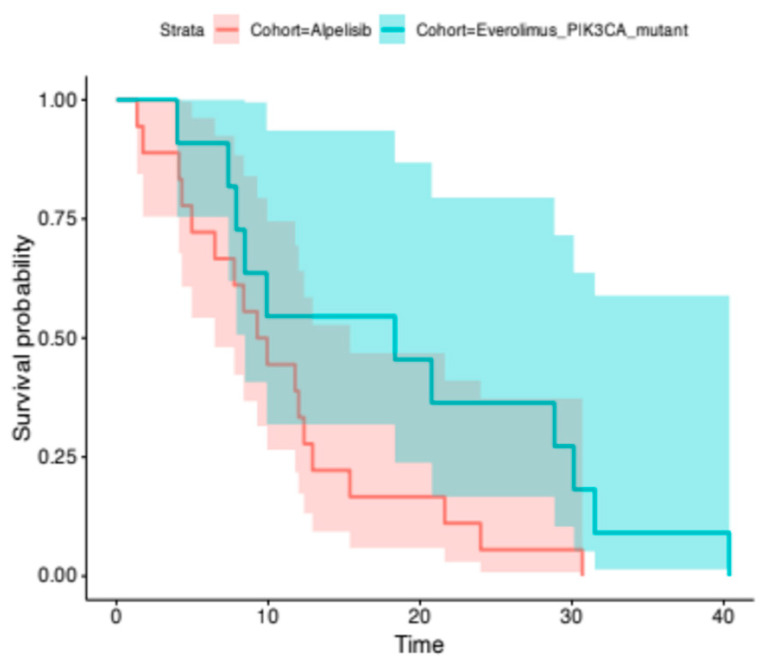
Kaplan–Meier analysis of the OS. The median OS for the alpelisib + ET group was 9.6 months (95% CI, 6.5–15.4), whereas the median OS was 18.3 months (95% CI, 8.4-NR) for the everolimus group (log-rank, *p*-value = 0.47).

At the time of analysis, the median PFS for the alpelisib + ET group was 4.9 months (95% CI, 3.4–9.5). The median PFS for the everolimus plus ET group was 4.5 months (95% CI, 2.8–6.7). The difference between the two groups was not statistically significant, as indicated by the Cox proportional hazards regression model (HR, 1.22; 95% CI, 0.65–2.28; *p*-value = 0.53) (Figure 1). The median OS for the alpelisib + ET group was 9.6 months (95% CI, 6.5–15.4), whereas the mOS for the everolimus group was 18.3 months (95% CI, 8.4-NR). Despite the numerical difference, the result was not statistically significant between the two groups (HR, 0.67; 95% CI, 0.25–1.76; *p*-value = 0.47) (Figure 2). After adjusting for baseline prognostic factors, including metastatic burden (single vs. >2 metastatic sites) and visceral disease, the treatment cohort was still not independently associated with OS. In an exploratory stratified analysis, median OS among patients with multiple metastatic sites and visceral disease ranged from approximately 9 months in the alpelisib group to 24 months in the everolimus group. On the other hand, median OS among patients with a single metastatic site and no visceral disease was very close for the two cohorts. However, these estimates were based on very small subgroups and should be interpreted as descriptive only. Further investigation is granted in the future for larger samples.

### 3.2. Comparison of Everolimus Plus ET in the PIK3CA-Mutant Population Versus Everolimus Plus ET in the PIK3CA-Wild Type Population

**Figure 3:**
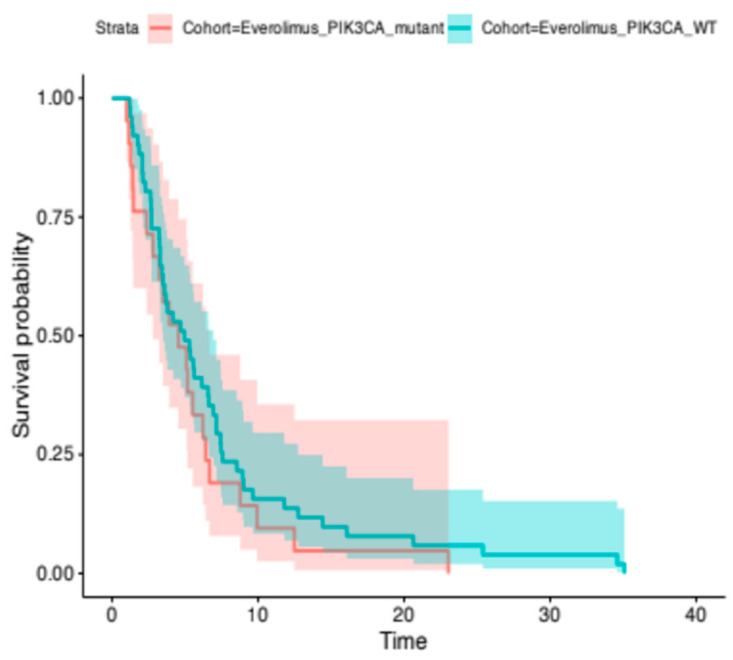
Kaplan–Meier analysis for median PFS. Median PFS in the *PIK3CA*-mutant everolimus plus ET group was 4.5 months (95% CI, 2.8–6.7) wheareas the median PFS was 5 months (95% CI, 3.5–6.9) for the PIK3CA-wild-type everolimus plus ET group (log-rank, *p*-value = 0.32).

Median PFS in the *PIK3CA*-mutant everolimus plus ET group was 4.5 months (95% CI, 2.8–6.7) compared to 5 months (95% CI, 3.5–6.9) for the PIK3CA-wild-type everolimus plus ET group. The difference was not statistically significant (HR, 0.77; 95% CI, 0.46–1.29; *p*-value = 0.32) (Figure 3). The median OS for the *PIK3CA*-mutant everolimus plus ET group was 18.3 months (95% CI, 8.4-NR), while for the *PIK3CA*-wild-type group, it was 13.7 months (95% CI, 11.1–17.3). Again, the difference between groups was not statistically significant (HR, 1.36; 95% CI, 0.66–2.78; *p*-value = 0.4).

### 3.3. Biopsy from the Primary Versus Metastatic Site (Alpelisib Group)

**Figure 4:**
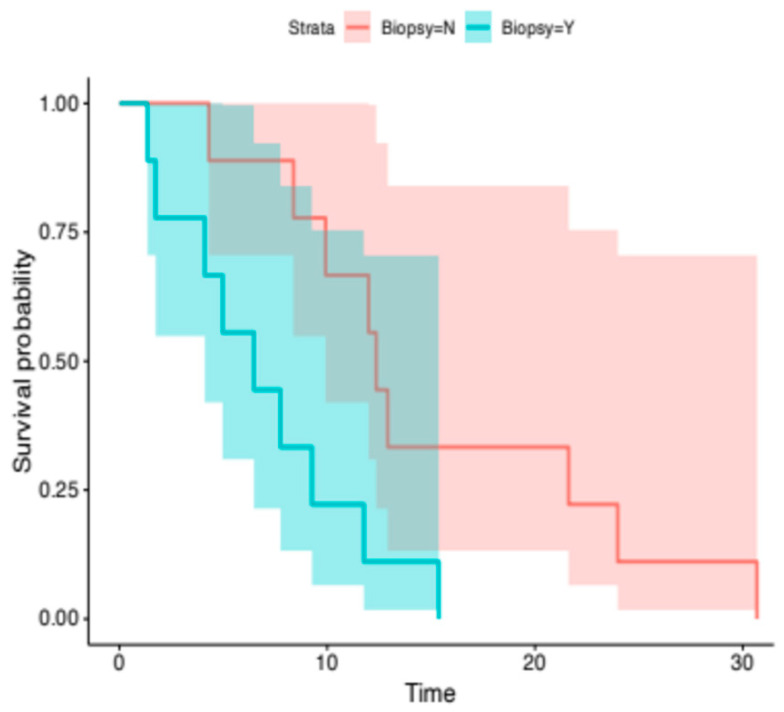
Kaplan–Meier analysis for OS. The median OS was 12.4 months (95% CI, 9.9-NR) for patients in whom PIK3CA mutation was determined through primary-site genomic analysis, compared with 6.5 months (95% CI, 4.1-NR) for patients in whom PIK3CA mutation was determined through metastatic-site genomic analysis (log-rank, *p*-value = 0.02).

*PIK3CA* mutations for 18 patients were determined based on molecular analysis of the primary site. For the remaining 22 patients, PIK3CA mutations were identified through the molecular analysis of the metastatic site. The median OS for patients in the former group was 12.4 months (95% CI, 9.9-NR) compared to 6.5 months (95% CI, 4.1-NR) for patients in the latter group. The difference between the two groups was statistically significant (HR, 3.5; 95% CI, 1.2–10.17; *p*-value = 0.02) (Figure 4).

### 3.4. Survival for Patients with <2 Metastatic Sites Versus ≥2 Metastatic Sites (Alpelisib Group)

**Figure 5:**
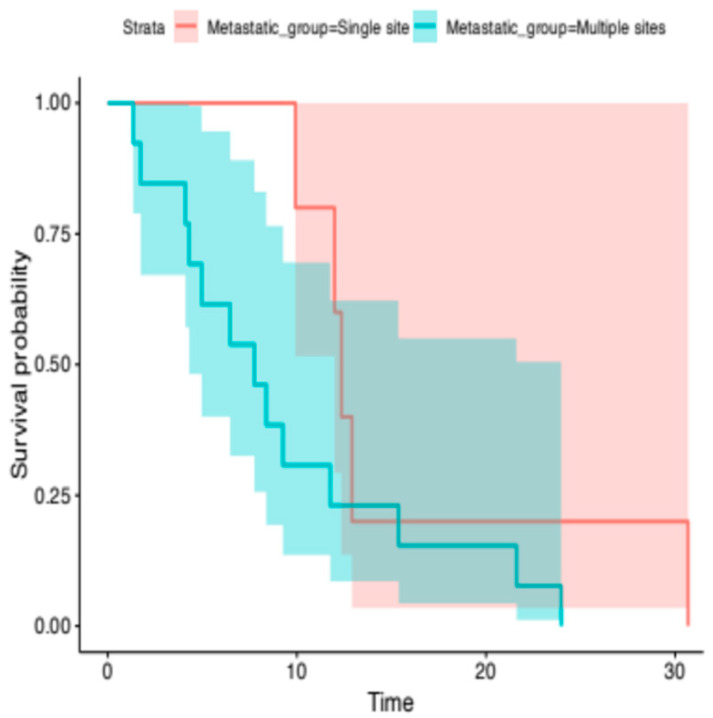
Kaplan–Meier analysis for OS. The median OS for the patients with <2 metastatic sites was 12.4 months (95% CI, 12-NR), compared to 7.4 months median OS (95% CI, 4.3-NR) for the group of patients with ≥2 metastatic sites (log-rank, *p*-value = 0.15).

Thirteen (13) patients had metastases located to <2 sites, while 27 patients had ≥2 metastatic sites. The median OS for the former group was 12.4 months (95% CI, 12-NR), whereas the median OS for the latter group was 7.4 months (95% CI, 4.3-NR). The difference between the two groups was not statistically significant (HR, 2.31; 95% CI, 0.74–7.21; *p*-value = 0.15) (Figure 5).

### 3.5. Response Rates for Alpelisib Plus ET and Everolimus Plus ET

The ORR was calculated for 29 (alpelisib group) and 19 patients (everolimus group), respectively. For eleven (11) patients in the former group and three (3) patients in the latter group, respectively, radiological assessment was not available, and they were excluded from the calculations. ORR was defined as the proportion of patients who achieved partial (PR) or complete response (CR) to therapy. In our population, none of the patients in either group achieved CR. Six (6), twelve (12), and eleven (11) patients in the alpelisib group had PR, stable disease (SD), and disease progression (PD), respectively. One (1), eleven (11), and seven (7) patients in the everolimus group had PR, SD, and PD, respectively. ORR was 20.7% (95% CI, 8–39.7%) for the alpelisib and 5.3% (95% CI, 0.13–26%) for the everolimus arm (Table 2). However, this difference is not statistically significant using Fisher’s exact test, yielding a *p*-value of 0.22.

### 3.6. Toxicity Profile

A total of 95% (38/40) of patients who received alpelisib experienced AEs of any grade, while 45% (18/40) disrupted the drug due to unacceptable toxicity. Most commonly observed AEs in the alpelisib group were hyperglycemia (23/40, 57.5%), rash (11/40, 27.5%), and anorexia (9/40, 22.5%). Grade ≥ 3 toxicity was noted in 18 patients (45%), and no death related to treatment (grade 5 toxicity) was observed. In the everolimus group 86.3% (19/22) of participants experienced AEs of any grade, and 18.2% (4/22) stopped drug due to toxicity. Most commonly, AEs were fatigue (9/22, 40.9%) and stomatitis (6/22, 27.3%). Grade ≥ 3 toxicity was observed in 7 patients (31.8%). Table 3 lists the observed AEs in the studied population. The AEs were graded according to Common Terminology for Adverse Events (CTCAE) version 5.0.

## 4. Discussion

To our knowledge, this is the first study directly comparing alpelisib in combination with ET versus everolimus plus ET after CDK4/6i progression in the metastatic setting. The median PFS for alpelisib group was 4.9 months compared to 4.5 months in the everolimus group, and the difference did not reach statistical significance (HR, 1.22; 95% CI, 0.65–2.28; *p* = 0.53). Conversely, the median OS was 18.3 months in the everolimus group, compared with 9.6 months in the alpelisib group. Despite the numerical difference, the result was not statistically significant (HR, 0.47; 95% CI, 0.21–1.08; *p* = 0.08). The ORR for the alpelisib arm was 20.7% (95% CI, 8–39.7%), which is inferior to the responses observed in SOLAR-1 (26.6%) and the BYLieve study (23%) [10,12].

In the literature, there is no robust evidence demonstrating the efficacy of alpelisib combined with fulvestrant post-CDK4/6i progression, and the data are mainly derived from retrospective studies. Cohort A of the BYLieve study is the only prospective, albeit non-comparative, cohort providing insights into survival outcomes [12]. High-level evidence is anticipated to be yielded by the EPIK-B5 trial (NCT05038735), an ongoing phase III study in which participants with metastatic HR+/HER2− BC are randomly assigned to receive alpelisib plus fulvestrant or placebo plus fulvestrant, provided that disease progresses on or after CDK4/6i. The trial is expected to present its initial results by 2026 [16]. The randomized phase II CAPTURE trial (ACTRN12619001117101) is another ongoing study assessing the combination of alpelisib and fulvestrant compared to capecitabine for patients who are progressing on or after CDK4/6i [17]. Regarding real-world data, the largest study to date included 233 consecutive patients from the French early access programme who received alpelisib in combination with fulvestrant. The median number of prior systemic therapies was four (4). Additionally, 97.4% of participants had previously been exposed to CDK4/6i, and 56.2% had received prior everolimus treatment. Reported median PFS was 5.3 months, median OS was 16.8 months, and the ORR was 38.8%. Subgroup analysis indicated that patients under 60 years of age and those who received more than 5 lines of treatment were adversely affected in terms of PFS [18].

Real-world evidence about the effectiveness of alpelisib in the post-CDK4/6 setting was also provided by Turner et al. In this retrospective study, the outcomes of 95 patients who received alpelisib in combination with fulvestrant were compared with those in the BYLieve trial. The median PFS and 6-month PFS of the real-world cohort were 3.7 months and 40.1%, respectively, which were inferior to the 7.3 months and 54.6% of the BYLieve study [19]. Alaklabi et al. published a single-center retrospective study of 27 patients who received alpelisib plus ET following CDK4/6i progression. The authors reported a median PFS of 6.8 months, while median OS was not reached. ORR was 12.5%. In this study, the exact ET partner(s) of alpelisib were not determined [20]. Studies that deal with the survival impact of alpelisib on patients who progressed on or after CDK4/6i therapy are listed in Table 4.

Regarding toxicity, in our survey, 18 patients (45%) in the alpelisib arm discontinued treatment due to adverse effects. In the alpelisib group, 38 out of 40 (95%), and in the everolimus group, 19 out of 22 (86.3%) experienced adverse events (AEs) of any grade. The most common AEs in the alpelisib arm were hyperglycemia (23/40, 57.5%), rash (11/40, 27.5%), and anorexia (9/40, 22.5%). Grade ≥ 3 toxicity was observed in 18 patients (45%). According to the authors of the SOLAR-1 trial, hyperglycemia, rash, and diarrhea are the AEs of special interest (AESI) of alpelisib, and exercise caution is needed for the prevention and management of these clinical situations. Hyperglycemia and rash tend to occur early after starting the drug, while diarrhea appears in a later stage [24]. In the SOLAR-1 trial, 99.3% of participants experienced AEs of any grade, with hyperglycemia (63.7%), diarrhea (57.7%), and nausea (44.7%) being the most common. Grade ≥ 3 AEs were reported in 76% of the alpelisib group, with 25% permanently discontinuing the drug due to toxicity [10]. In the BYLieve study, the most frequently reported AEs were diarrhea (65%), hyperglycemia (60%), and nausea (46%), with toxicity-related discontinuation reaching up to 23% [12]. In a real-world setting, hyperglycemia, rash, and fatigue were among the most common AEs, as demonstrated by the French retrospective study [18]. These findings align with a post-marketing survey conducted by the World Health Organisation (WHO) [25]. Furthermore, in real-world practice, discontinuation rates due to unacceptable toxicity are higher than those observed in the SOLAR-1 and BYLieve studies, ranging up to 40% (Table 4). Hyperglycemia is a significant AE of alpelisib treatment. Despite that, there are well-established risk factors for the occurrence of hyperglycemia; individuals without a history of diabetes or any other comorbidities could also manifest this issue [26]. The *PI3K*/*AKT*/*mTOR* pathway masters the transcription of glycolytic enzymes, and its drug-induced inhibition results in the impairment of innate insulin balance, glycose transportation, and metabolism [27]. The METALLICA trial was a phase II, 2-cohort, single-arm study that enrolled patients with *PI3KCA*-mutant metastatic breast cancer who received metformin at the initiation of treatment with alpelisib. Cohort A included normoglycemic patients, while cohort B included patients with prediabetes. One patient in cohort A and three in cohort B developed grade ≥ 3 hyperglycemia, respectively. 13.2% of the participants discontinued alpelisib due to toxicity, albeit none attributable to impaired glucose levels [28]. Based on these results, the prophylactic use of metformin represents an efficacious method for preventing alpelisib-induced hyperglycemia, and this practice has also been incorporated into our therapeutic protocol.

Despite the wide approval of alpelisib, in many countries, it is either unavailable or healthcare systems have recommended against its reimbursement [29]. National Comprehensive Cancer Network (NCCN) and European Society of Medical Oncology (ESMO) guidelines recommend the use of everolimus, an *mTOR* inhibitor, which could be utilized regardless of the mutational status of the tumor. The BOLERO-2 trial validated the efficacy of everolimus plus the steroidal AI exemestane, but in the pre-CDK4/6i era [13]. In the CDK4/6i era, no randomized trials have addressed the impact of everolimus, and the data are mainly coming from small retrospective studies. Recently, Vasseur et al. published a prospective, non-comparative study that provided evidence on the efficacy of everolimus as a subsequent therapy after CDK4/6i failure. Notably, the authors used fulvestrant as an ET partner rather than exemestane, as in the BOLERO-2 trial. Median PFS and OS were 6.8 and 38.2 months, respectively [15]. Mo H. et al. conducted a retrospective study of 79 patients treated with everolimus plus exemestane post-CDK4/6 progression. Median PFS was 3.8 months, while median OS was 22.6 months [30]. Studies about the effectiveness of everolimus plus ET after CDK4/6i failure are listed in Table 5.

The impact of everolimus on *PIK3CA*-mutant tumors has not been investigated in the CDK4/6i era. The subgroup analysis of 25 patients whose tumors harbored *PIK3CA* mutations in the Vasseur et al. study showed a median PFS of 3.4 months and a median OS of 22.9 months [15]. In our analysis, patients with *PIK3CA*-mutant metastatic BC achieved median PFS and OS of 4.5 and 18.3 months, respectively, while patients without *PIK3CA* mutations exhibited median PFS and OS of 5.5 and 13.4 months, respectively. The correlative analysis of the genomic alterations in the BOLERO-2 trial revealed that the *PIK3CA*-mutant subgroup demonstrated a median PFS of 6.8 months, whereas the *PIK3CA*-wild-type subgroup had a median PFS of 8.8 months [14]. It is again highlighted that the BOLERO-2 trial results were obtained before the CDK4/6i era. The difference in OS between *PIK3CA*-mutant and *PIK3CA*-wild-type tumors in our analysis could not be merely explained by the presence of one mutation. Other mutations with known (e.g., *ESR1*) and unknown contribution to the natural history of the disease may play a role, as well as other parameters such as tumor burden and prior therapies.

In our survey, we investigated whether the determination of *PIK3CA* status from either the primary or metastatic site has an impact on survival. Patients whose *PIK3CA* mutational status was established in the primary tumor genomic analysis had a median OS of 12.4 months compared to 6.5 months for those whose *PIK3CA* mutation was identified in the metastatic site, and this difference was statistically significant (HR, 3.5; 95% CI, 1.20–10.17; *p* = 0.02). One plausible explanation is the fact that 61.1% (11/18) of patients in the first group had metastatic disease located in a single organ, and more specifically, 55.5% (10/18) had bone-only disease. In contrast, only 9.1% (2/22) of patients in the latter group had single-organ metastatic disease. Metastatic disease manifesting as bone-only disease seems to have a more quiescent natural history with more favorable outcomes [33]. Moreover, as demonstrated in our study, patients with single-organ metastasis have better survival outcomes compared to those with multiple metastatic sites (OS, 12.4 months vs. 7.8 months, *p*-value = 0.15). A bone biopsy is often technically challenging, and bone does not represent an ideal tissue for executing genomic analysis. Therefore, in cases of bone-only metastatic disease, primary tissue examination is the only option for obtaining genomic information. Nevertheless, in recent years, ctDNA has gained ground as a valid alternative for tracking actionable mutations [34]. It is also important to note that discordance in *PIK3CA* mutational status between the primary and metastatic site(s) is evident in approximately 10% of cases [35]. Hence, the absence of mutation in the primary tumor does not preclude the absence of mutation in any metastatic site or vice versa.

Our study has limitations that need to be addressed. Firstly, the retrospective design makes it susceptible to information and selection bias. Additionally, the small number of participants in each group limits the robustness of the results. Our survival outcomes with alpelisib were worse compared to those reported in the literature. Possible reasons include the heterogeneity of the population, with 30% of participants receiving chemotherapy before alpelisib, and 40% receiving ET other than fulvestrant. Furthermore, 45% of the population permanently discontinued the drug due to AEs, while nearly all patients (95%) experienced toxicity requiring multiple drug interruptions, lasting over 3 days, especially for grade ≥ 3 AEs. Since the half-life of alpelisib ranges between 8 and 9 h, prolonged interruptions can reduce its therapeutic potential [36]. A retrospective study by Cheung et al. demonstrated that shorter exposure to alpelisib (<90 days) is associated with poorer survival outcomes [37]. Moreover, the numerical difference in OS between alpelisib and everolimus could be attributed to differences in the two groups’ characteristics. In the everolimus group, more patients had bone-only disease than in the alpelisib group, and fewer patients had high-burden disease (≥2 metastatic sites). A multivariate analysis was not provided due to the very small sample size. Therefore, we could not further analyze whether the difference between alpelisib and everolimus was driven by the above imbalances.

One important drawback of our study is the lack of utilization of ctDNA assays. CfDNA tumor fraction is generally elevated in a metastatic setting, rendering ctDNA a valuable and accurate tool for the detection of actionable mutations [38]. Using ctDNA, we could potentially detect more patients with *PIK3CA* mutations, particularly those with an unknown mutational profile due to inadequate primary tumor samples and difficulties in obtaining biopsies from a metastatic site. Moreover, a tissue biopsy only reveals the mutational status of the specific site from which it was obtained; therefore, it does not preclude the existence of a specific mutation. Conversely, ctDNA could reveal the clonality of the disease and its spatial and temporal heterogeneity [39]. Notably, based on the SOLAR-1 trial, the Therascreen^®^ PIK3CA RGQ PCR Kit (QIAGEN N.V., Venlo, The Netherlands) was the first companion diagnostic (CDx) test approved by the FDA for the detection of PIK3CA mutations in patients with metastatic breast cancer [10]. Therascreen^®^ detects up to 11 different *PIK3CA* mutations covering approximately 70% of all known types of *PIK3CA* mutations. Hence, ctDNA assays with high-throughput genomic coverage could reveal additional mutations, increasing the number of patients eligible for alpelisib [40]. Another significant advantage of ctDNA is the rapid turnaround time for results. Obtaining tissue results is usually a time-consuming process, and we believe that using a ctDNA assay could render a significant percentage of patients eligible to receive alpelisib immediately after CDK4/6i failure, thereby sparing the need for chemotherapy. *ESR1* mutations are a well-known mechanism of resistance to AI therapy, and ESMO guidelines strongly endorse ctDNA as the preferred diagnostic tool for detecting *ESR1* mutations in the metastatic setting [41]. In our cohort, 12.5% (5/40) of patients had mutations in both *PIK3CA* and *ESR1*. This is a distinctive subgroup with few existing data about the effectiveness of *PIK3CA* targeted therapy in combination with ET, provided that the *ESR1*-mutant tumors could also benefit from the use of oral SERD, elacestrant [42]. The use of a ctDNA assay could detect more patients with dual mutations, enabling us to provide evidence regarding this subgroup.

## 5. Conclusions

Our results regarding the efficacy of alpelisib after CDK4/6i progression did not align with the existing literature, and its toxicity profile remains challenging. On the other hand, we demonstrated that everolimus represents a valid alternative in patients with *PIK3CA*-mutant metastatic BC. The numerical difference in OS between alpelisib and everolimus can be explained by the lower metastatic burden in the latter group (<2 metastatic sites: 59.1% vs. 32.5%), the highest rate of bone-only disease (45.5% vs. 25%), and the fact that 77.3% of patients in the everolimus group were exposed only to one treatment line compared to 62.5% in the alpelisib group. The field is rapidly evolving, and newer therapies targeting the *PI3K*/*AKT*/*mTOR* pathway have emerged. Inavolisib is a selective inhibitor and degrader of the p110α subunit of PI3K protein. It has been approved as a first-line treatment in combination with the CDK4/6i agent palbociclib and fulvestrant [43]. Capivasertib, a small-molecule inhibitor of all three AKT isoforms, has also been approved in combination with fulvestrant for tumors harboring mutations in *PIK3CA*, *AKT* or *PTEN* [44]. The selection of the optimal therapeutic regimen in *PIK3CA*-mutant metastatic BC is a chess-like strategic process, and more evidence is needed to elucidate all the parameters to be considered.

## Figures and Tables

**Table 1:** Study’s demographics.

Characteristics	Alpelisib (*n* = 40)	Everolimus (*PIK3CA*-Mutant) (*n* = 22)
**Age (median)**	63	62.4
**<65-no. (%)**	22 (55)	12 (54.5)
**≥65-no. (%)**	18 (45)	10 (45.5)
**Performance status-no. (%)**		
**0**	35 (87.5)	19 (86.4)
**1**	5 (12.5)	3 (13.6)
**2**	-	-
**Number of metastatic sites-no. (%)**		
**<2 sites**	13 (32.5)	13 (59.1)
**≥2 sites**	27 (67.5)	9 (40.9)
**Bone-only disease-no. (%)**		
**Yes**	10 (25)	10 (45.5)
**No**	30 (75)	12 (54.5)
**Visceral disease-no. (%)**		
**Yes**	23 (57.5)	11 (50)
**No**	17 (42.5)	11 (50)
**Site of molecular analysis-no. (%)**		
**Primary tumor**	22 (55)	8 (36.4)
**Metastatic site**	18 (45)	14 (63.6)
**Prior treatment line(s)-no. (%)**		
**=1**	25 (62.5)	17 (77.3)
**>1**	15 (37.5)	5 (22.7)
**Prior chemotherapy (for stage IV)-no. (%)**		
**Yes**	12 (30)	5 (22.7)
**No**	28 (70)	17 (77.3)
**Detected *PIK3CA* mutation(s)-no. (%)**		
**Single**	34 (85)	20 (90.9)
**Double**	6 (15)	2 (9.1)
**ET partner-no. (%)**		
**Fulvestrant**	24 (60)	-
**Non-steroidal AI**	2 (5)	-
**Steroidal AI**	12 (30)	22 (100)
**Tamoxifen**	2 (5)	-

Abbreviations: AI, aromatase inhibitors; ET, endocrine therapy.

**Table 2:** Response rates.

Best Response	Alpelisib (*n* = 29)	Everolimus (*n* = 19)
**CR-no. (%)**	-	-
**PR-no. (%)**	6 (20.7)	1 (5.3)
**SD-no. (%)**	12 (41.4)	11 (57.9)
**PD-no. (%)**	11 (37.9)	7 (36.8)
**ORR**		
**No. of patients**	6	1
**% of patients (95% CI)**	20.7 (8–39.7)	5.3 (0.13–26)

Abbreviations: CR, complete response; ORR, objective response rate; PD, progression disease; PR, partial response; SD, stable disease.

**Table 3:** Toxicity profile of the studied population.

Event	Alpelisib		Everolimus	
	(*n* = 40) (%)		(*n* = 22) (%)	
	Any	Grade ≥ 3 *	Any	Grade ≥ 3 *
**Hyperglycemia**	23 (57.5)	10 (40)	3 (13.6)	-
**Rash**	11 (27.5)	6 (15)	3 (13.6)	-
**Anorexia**	9 (22.5)	1 (2.5)	2 (9.1)	-
**Diarrhea**	7 (17.5)	1 (2.5)	2 (9.1)	1 (4.5)
**Nausea**	7 (17.5)	3 (7.5)	2 (9.1)	-
**Fatigue**	6 (15)	1 (2.5)	9 (40.9)	1 (4.5)
**Vomiting**	4 (10)	2 (5)	2 (9.1)	-
**Pruritus**	4 (10)	-	1 (4.5)	-
**Stomatitis**	2 (5)	-	6 (27.3)	2 (9.1)
**Alopecia**	2 (5)	-	-	-
**Dysgeusia**	2 (5)	-	1 (4.5)	-
**Arthralgia**	2 (5)	-	2 (9.1)	-
**Headache**	1 (2.5)	-	3 (13.6)	1 (4.5)
**Anemia**	-	-	3 (13.6)	1 (4.5)
**Pneumonitis**	-	-	3 (13.6)	-
**Cough**	-	-	3 (13.6)	-
**Peripheral edema**	-	-	2 (9.1)	-
**Hypertriglyceridemia**	-	-	2 (9.1)	1 (4.5)
**Constipation**	-	-	1 (4.5)	-
**Thrombocytopenia**	-	-	1 (4.5)	-

* No Grade 5 toxicity.

**Table 4:** Studies investigated the impact of alpelisib post-CDK4/6i progression.

Study’s First Author, Year of Publication [Reference]	Study Design	No. of Participants	PFS/OS	RR (%)	ET Partner	Discontinuation Rate (%) *
**Rugo H.,** **2024 [12]**	Phase II, single-arm	127	8/27.3 (months)	23	Fulvestrant	23
**Roufai D.,** **2023 [18]**	Retrospective	233	5.3/16.8 (months)	38.8	Fulvestrant	39.1
**Turner S.,** **2021 [19]**	Retrospective	95	3.7/NA (months)	NA	Fulvestrant	20.5
**Alaklabi S., 2022 [20]**	Retrospective	27	6.8/NR (months)	12.5	NA	40.7
**Miller J.,** **2022 [21] ^†^**	Retrospective	15 18	6.2/200 5.5/154 (months/days)	NA	Fulvestrant	27
**Raphael. A, 2022 [22] ^‡^**	Retrospective	12	5.5/NA (months)	NA	Fulvestrant, Exemestane	NA
**Morrison L., 2025 [23]**	Retrospective	51	NA/11 (months)	29	NA	19

Abbreviations: ET, endocrine therapy; NA, not available; NR, not reached; OS, overall survival; PFS, progression-free survival; RR, response rate. * Discontinuation rate due to alpelisib’s adverse events. ^†^ The authors reported results from 2 cohorts. One which included patients who received ≤2 treatment lines before alpelisib, and the other that included patients who received ≥3 treatment lines before alpelisib. ^‡^ The authors reported that all patients received everolimus prior to alpelisib.

**Table 5:** Studies investigated the effect of everolimus plus ET after CDK4/6i progression.

(A) Efficacy of Everolimus + ET After CDK4/6i Progression (Regardless *PIK3CA* Mutation)					
Study’s First Author, Year of Publication [Reference]	Study Design	No. of Participants	PFS/OS	RR (%)	ET Partner
**Mo, H.,** **2022 [30]**	Retrospective	79	3.8/22.6 (months)	NA	Exemestane
**Xi, J.,** 2020 [31] *	Retrospective	12	4.9/NA (months)	NA	Exemestane
**Dhakal, A.,** **2020 [32]**	Retrospective	41	4.2/18.7 (months)	17.1	NA
**Vasseur, A.,** **2024 [15]**	Prospective, single-arm	57 25	6.8/38.2 (months) 3.4/22.9 (months) ^†^	31.9 NA	Fulvestrant

Abbreviations: ET, endocrine therapy; NA, not available; OS, overall survival; PFS, progression-free survival; RR, response rate. * Subgroup analysis. ^†^ Subgroup analysis of patients with *PIK3CA*-mutant metastatic BC.

## Data Availability

The data presented in this study are available from the corresponding author upon request, due to ethical considerations.
